# Acute hyperthermia and hypoxia tolerance of two improved strains of nile tilapia (*Oreochromis niloticus*)

**DOI:** 10.1007/s44154-023-00099-w

**Published:** 2023-06-29

**Authors:** Kwasi Adu Obirikorang, Richard Appiah-Kubi, Daniel Adjei-Boateng, Wonder Sekey, Collins Prah Duodu

**Affiliations:** 1 Department of Fisheries and Watershed Management, University Post Office, Kwame Nkrumah University of Science and Technology, Private Mail Bag, Kumasi, Ghana; 2 Department of Marine and Fisheries Sciences, University of Ghana, Accra, Ghana

**Keywords:** Aquaculture, Aquatic hypoxia, Environmental stressors, Oxygen, Temperature

## Abstract

Tilapia production in Ghana has been hit with episodes of stress and pathogen-induced mass fish kills which have anecdotally been linked to the culture of illegally imported Genetically Improved Farmed Tilapia (GIFT) strains of Nile tilapia, *Oreochromis niloticus*. This study was thus set up to comprehensively assess the stress tolerance of the GIFT strain and a native strain of Nile tilapia (the Akosombo strain) following exposures to hyperthermic and hypoxic stressors. In a series of experiments, oxygen consumption (*M*O_2_), aquatic surface respiration (ASR), thermal limits and hypoxia tolerance were assessed. The effects of these stressors on haematological parameters were also assessed. The GIFT strain was less tolerant of hypoxia and performed ASR at higher O_2_ levels than the Akosombo strain. Under progressive hypoxia, the GIFT strain exhibited higher gill ventilations frequencies (*f*V) than the Akosombo strain. The thermal tolerance trial indicated that the Akosombo strain of *O. niloticus* has higher thermotolerance than the GIFT strain and this was reflective in the higher LT_50_ (45.1℃) and LT_max_ (48℃), compared to LT_50_ and LT_max_ of 41.5℃ and 46℃ respectively. These results imply that it is crucial to consider how the GIFT strain performs under various environmental conditions and changes during culture. Particularly, raising the GIFT strain of Nile tilapia in earthen ponds rich in phytoplankton and subject to protracted episodes of extreme hypoxia may have a detrimental physiological impact on its growth and welfare.

## Introduction

Globally, the aquaculture production of Nile tilapia (*Oreochromis niloticus*) has expanded from 1 million to 4.4 million tonnes annually over the past two decades (FAO 2022). It is the third most cultured freshwater species behind the grass and silver carps (FAO 2022). The rapid expansion of *O. niloticus* production has been boosted by the WorldFish Centre’s selective breeding programme, the Genetically Improved Farmed Tilapia (GIFT), which was initiated in the late 1980s (Yáñez et al. 2020). The GIFT strain was produced by crossing four wild strains sourced from four African countries, including Ghana, and four farmed strains from the Philippines (Ponzoni et al. 2011). The faster-growing GIFT strain of *O. niloticus* typically grows 85% larger than the stock used at the beginning of the breeding program (WorldFish 2015). The subsequent distribution of the GIFT strain for commercial aquaculture now forms the backbone of *O. niloticus* culture in more than 87 countries across Africa, Asia, and South and Central America, and has revolutionised the expansion of the global production of tilapia (Eknath et al. 1993; Teletchea 2019; Tran et al. 2021). The GIFT strains were, however, not disseminated to the four African countries where the parent stocks were sourced due to the risks of genetic contamination of the locally adapted native stocks (Gupta and Acosta 2004; Anane-Taabeah et al. 2019; Ragasa et al. 2022a). Instead, WorldFish supported these countries in developing their own fast-growing *O. niloticus* strains through selective breeding programs (Trinh et al. 2021).

In 2001, WorldFish partnered with the Water Research Institute of Ghana to improve the local strain of Nile tilapia to replicate the productivity success previously achieved in Asia (Water Research Institute 2013). The fish seeds presently used in Ghanaian aquaculture have also undergone several generations of selective breeding from a base population sourced from three middle and northern sections of the Volta River basin and a fish farm in the southern part of the country (Attipoe et al. 2013). After two decades of selective breeding, the Akosombo strain (Ghana’s selectively-bred *O. niloticus* strain) grows 30% faster than non-improved farmed tilapia strains and has shortened culture periods from eight to six months (Trinh et al. 2021). Currently, it is the only *O. niloticus* strain permitted for use in local aquaculture production, restricting the importation of other improved strains except for research purposes (Ragasa et al. 2022b). To evaluate the growth improvements of the Akosombo strain, a controlled comparative growth trial was conducted by the country’s Aquaculture Research Development Centre (ARDEC) in 2012 using an imported GIFT strain as a control (Trinh et al. 2021; Ragasa et al. 2022b). On average, the GIFT strain grew twice as fast as the Akosombo strain and had better feed conversion efficiencies (Trinh et al. 2021). Consequently, many commercial fish farmers in Ghana have expressed discontentment with the growth performance of the Akosombo strain leading to illegal importations of some GIFT strains into the country for culture (Anane-Tabeah et al. 2019). Recent genetic studies (Anane-Taabeah et al. 2019; Addo 2021; Anane-Taabeah et al. 2022) have confirmed the use of mixed broodstocks derived from combinations of pure Akosombo strains and several other derivatives of the GIFT strain in many hatcheries in Ghana, despite the Akosombo strain being the only permitted *O. niloticus* strain for culture.

Environmental parameters such as temperature and oxygen levels significantly affect the physiological states of fish and can directly induce stress outside their optimum ranges for fish. Temperature is also one of the most important environmental factors related to disease outbreaks. For example, the epidemiological issues associated with the common pathogens in aquaculture are generally exacerbated during the warmer months, confirming their temperature-dependent pathogenicity (Ramírez‐Paredes et al. 2021). In addition to water temperature, hypoxia is also considered a crucial environmental factor affecting fish welfare and health, especially in aquaculture ponds that experience severe respiration-induced hypoxic episodes for several hours daily (Obirikorang et al. 2020). There is a general perception among fish farmers in Ghana that the GIFT strains are more susceptible to these environmental stressors and disease conditions, despite their superior growth rates. Besides the possibility of the illegally-sourced GIFTs introducing foreign pathogens into the country, there are other concerns that the GIFT strain will have poorer resistance to pathogens that the local strains, over time, have evolved resistance against (Ansah et al. 2014). The poor resistance of the GIFT strain of Nile tilapia to environmental stressors was confirmed by Sifa et al. (2002), who evaluated hypothermia tolerance in three different strains of Nile tilapia and observed that the GIFT strain was less tolerant than native strains. Anecdotally, the epidemiological issues and mass fish kills in Ghana’s aquaculture over the past eight years have been associated with the introductions of GIFT strains. More recently, episodes of hypoxia-induced mass fish kill at a large-scale cage farm in southern Ghana were linked to the culture of GIFT strains. These massive mortalities over a very short period were attributed to abrupt depletions in dissolved oxygen levels coupled with high temperatures. Nile tilapia under culture conditions are sometimes subjected to respiration-induced episodes of nocturnal hypoxia that can be severe (O_2_ levels < 1 mgL^−1^) (Obirikorang et al. 2020). Depending on the severity of oxygen decline, Nile tilapia can cope with these behaviourally by increasing gill ventilation frequencies or by engaging in aquatic surface respiration (ASR) at the air–water interface. Physiologically, the Nile tilapia like most teleosts, also increase counts of red blood cell parameters under hypoxic conditions to enhance oxygen uptake (Sheng et al. 2019). This study was thus set up to comprehensively examine the tolerance limits of the native and GIFT strains of Nile tilapia following exposures to hyperthermic and hypoxic stressors. It also assessed the effects of these stressors on some haematological parameters and adaptive behavioural responses in the two strains of Nile tilapia.

## Results

### Oxygen Consumption Rate

**Fig. 1:**
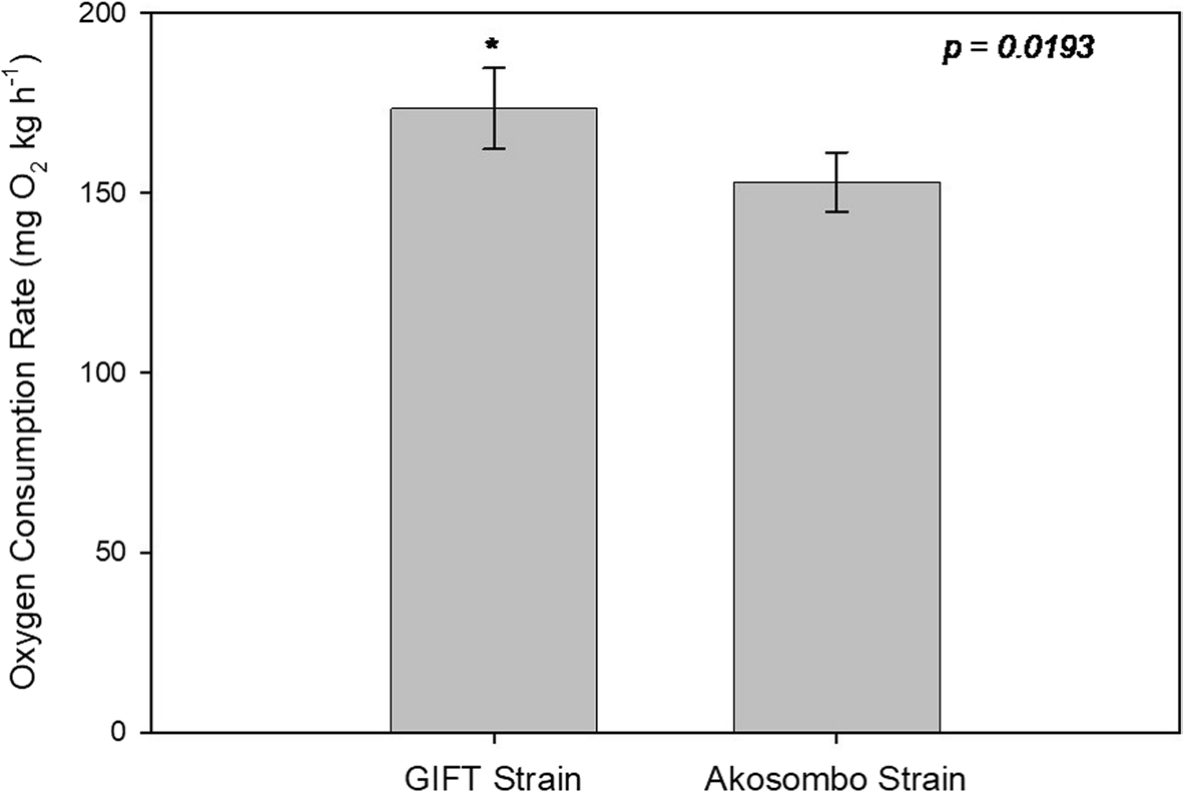
Oxygen consumption rates (*MO*_*2*_) of the GIFT and Akosombo strains of Nile tilapia. Each bar is the mean ± SD from a triplicate group. Asterisk indicates a significantly higher *MO*_*2*_ in the GIFT group relative to the Akosombo group

A remarkable strain effect on O_2_ demand was observed during the O_2_ consumption phase of the Oxygen consumption rates (*MO*_*2*_) trials. The O_2_ declines were steeper for the GIFT strain than the Akosombo strain. The calculated *MO*_*2*_ response of the GIFT strain (173.53 ± 11.32 mg O_2_ kg^−1^ h^−1^) was significantly higher (*p* = 0.0193) than the *MO*_*2*_ of the Akosombo strain (153.07 ± 8.30 mg O_2_ kg^−1^ h^−1^) (Fig. 1). The difference in *MO*_*2*_ responses of fasted groups of the two strains was 13%.

### Aquatic Surface Respiration

**Fig. 2:**
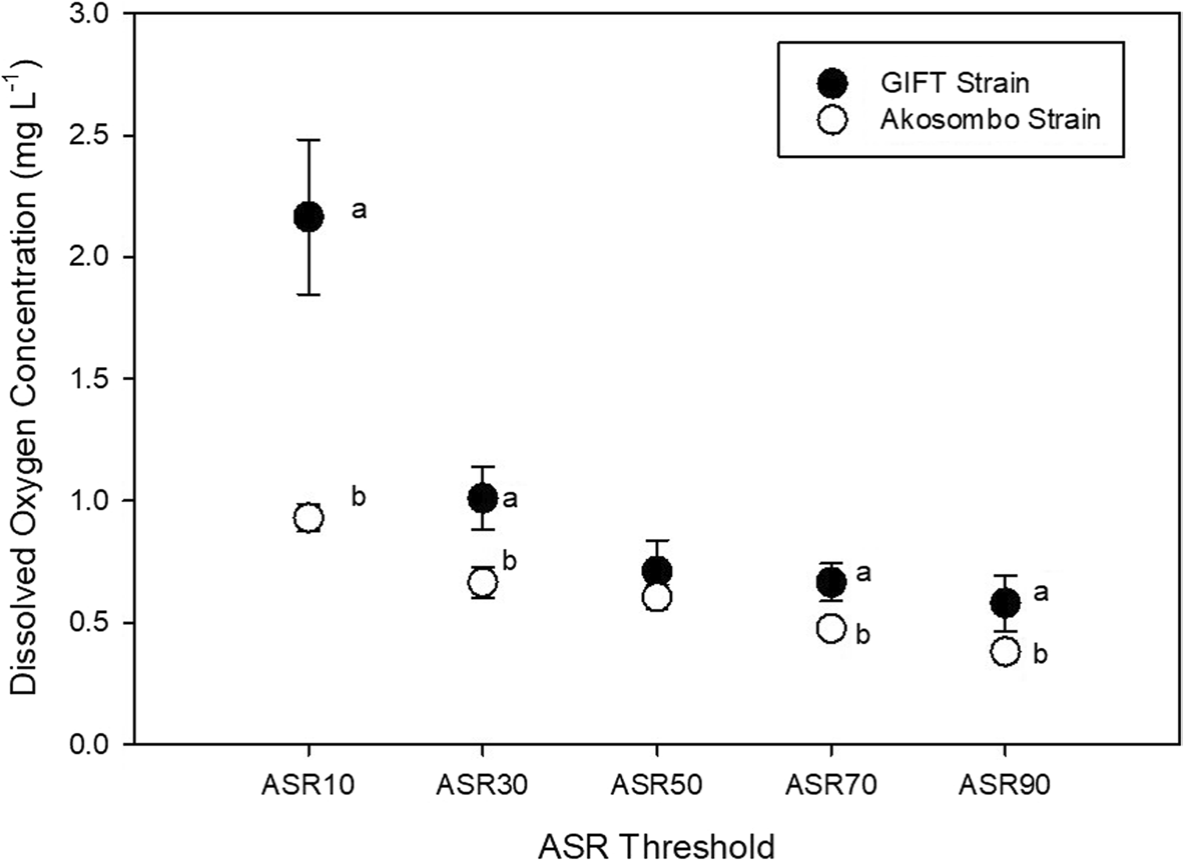
Aquatic surface respiration (ASR) thresholds of the GIFT and Akosombo strains of Nile tilapia. Lower case letters indicate significant differences (*p* < 0.05) among treatment groups at each ASR threshold

There were strain-specific differences in the thresholds at which Aquatic Surface Respiration (ASR) was employed. The GIFT strain, in most instances, was less tolerant of hypoxia and performed ASR at higher O_2_ levels than the Akosombo strain (Fig. 2). During the progressively induced hypoxia (from 6.5 to 0.2 mg L^−1^), the GIFT strain first employed ASR (ASR_10_) at a significantly higher (*p* < 0.0001) O_2_ threshold of 2.17 ± 0.32 mg L^−1^ compared to the Akosombo strain which utilised ASR at a threshold of 0.93 ± 0.05 mg L^−1^. At all the other ASR thresholds, the Akosombo strain tolerated significantly lower O_2_ levels than the GIFT strain before performing ASR. The O_2_ threshold at which ASR_90_ occurred for the Akosombo strain (0.38 ± 0.04 mg L^−1^) was 34% lower than the threshold for the GIFT strain (0.58 ± 0.11 mg L^−1^).

### Gill Ventilation Frequency

**Fig. 3:**
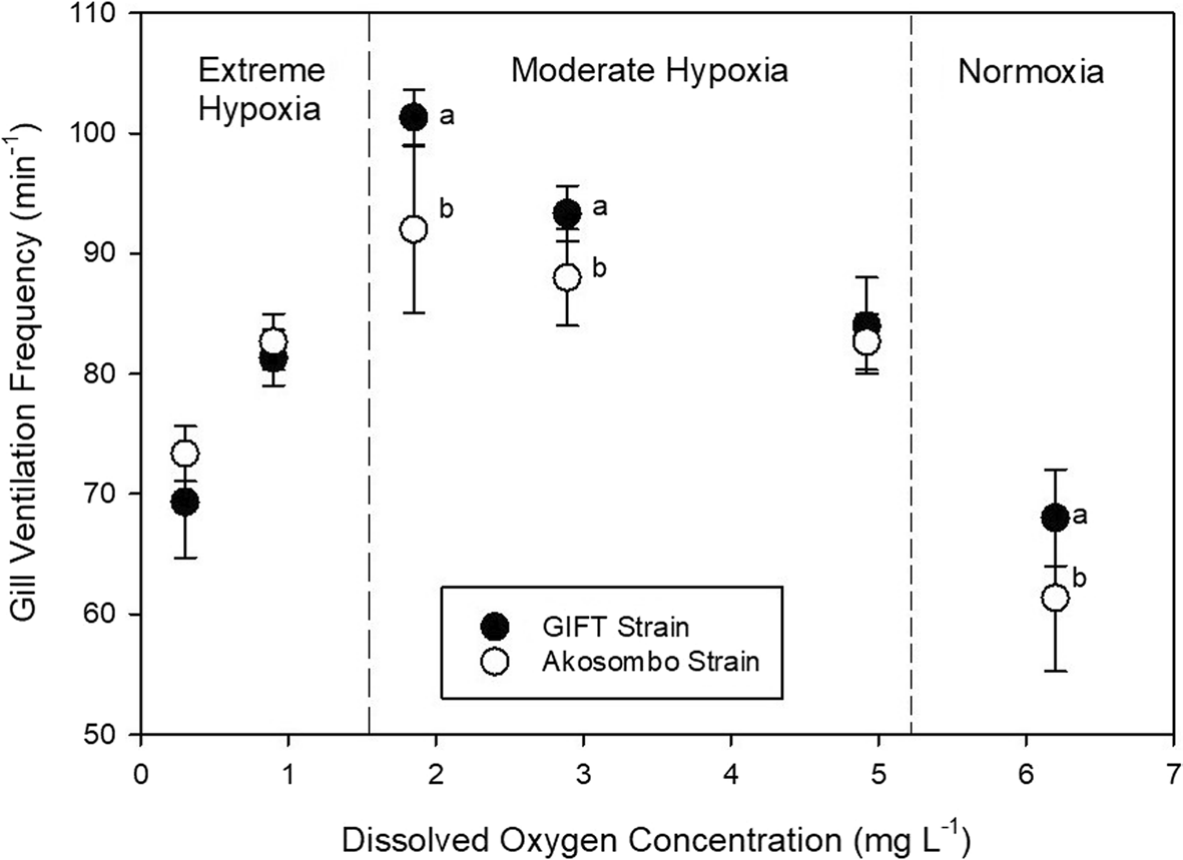
Ventilation frequencies of the Akosombo and GIFT strains of Nile tilapia adults at different dissolved oxygen concentrations. Treatment means at each dissolved oxygen concentration with different letters are significantly different from each other (*p* < 0.05)

**Fig. 4:**
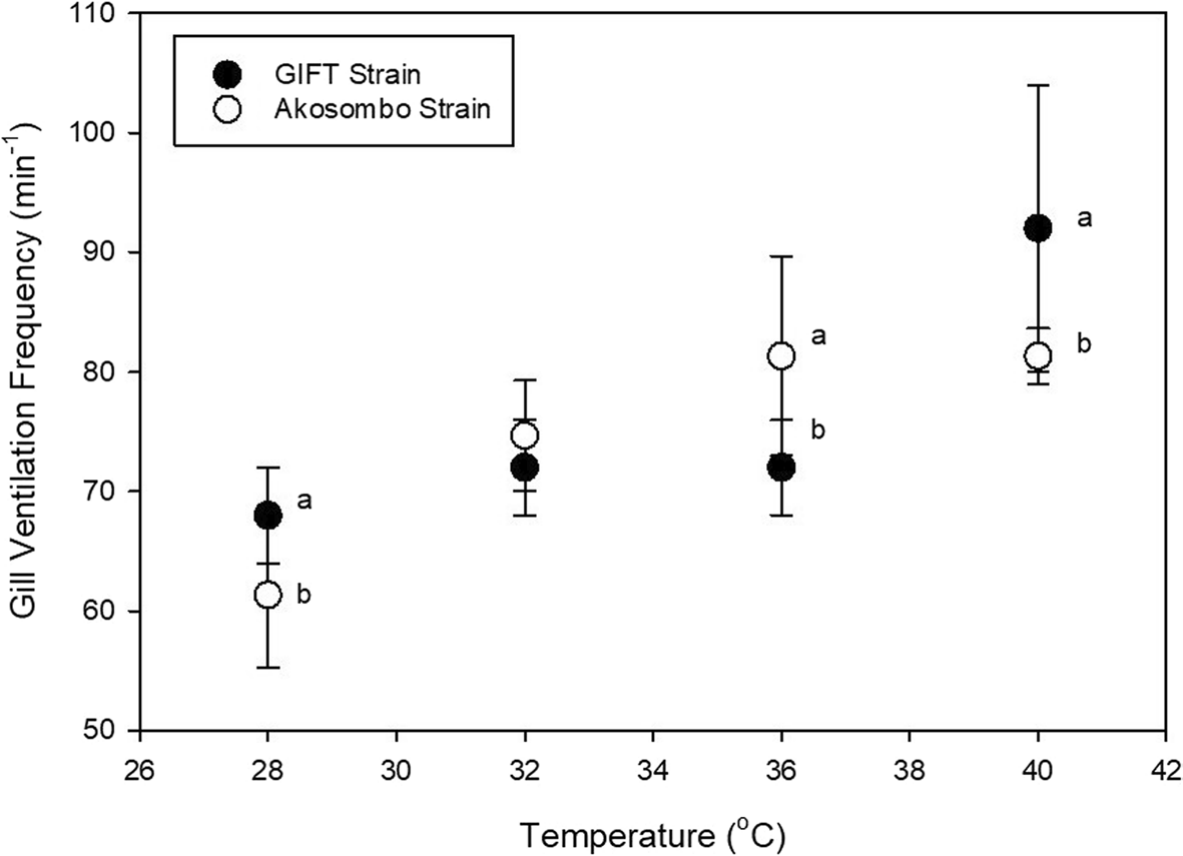
Ventilation frequencies of the Akosombo and GIFT strains of Nile tilapia adults at different water temperatures. Treatment means at each dissolved oxygen concentration with different letters are significantly different from each other (*p* < 0.05)

Hypoxia-mediated elevations in gill ventilation frequencies were observed in the two strains. Ventilation frequencies increased linearly with declining O_2_ levels up to an acute hypoxia threshold. However, individuals of the Akosombo strain, exhibited significantly lower *f*V than the GIFT strains. Under normoxic conditions, before the initiation of hypoxia, the GIFT strain had significantly higher (*p* < 0.05) mean fV (68.0 ± 4.0 beats min^−1^) compared to the Akosombo strains (61.3 ± 6.1 beats min^−1^). Hyperventilatory responses for both groups peaked at an O_2_ threshold of 1.8 mg L^−1^ below which *f*Vs decreased for both groups of fish (Fig. 3). Peak *f*V of the Akosombo and GIFT strains corresponded to 92.0 ± 6.9 and 101.3 ± 2.3 beats min^−1^, respectively. Mean *f*Vs following the critical O_2_ level decreased between 20 and 32% for the GIFT strain and 9 and 20% for the Akosombo strain. Increasing temperatures from 28–40 ℃ linearly increased the *f*Vs for both strains (Fig. 4). Relative to baseline *f*Vs at 28 ℃, the *f*Vs for the Akosombo and GIFT strains at 40 ℃ increased by 33 and 35%. At 40 ℃, the GIFT strain had significantly higher *f*Vs (*p* < 0.05) than the Akosombo strain. Within the temperature increment window, none of individuals of the two strains exhibited depressions in *f*V.

### Haematology

**Fig. 5:**
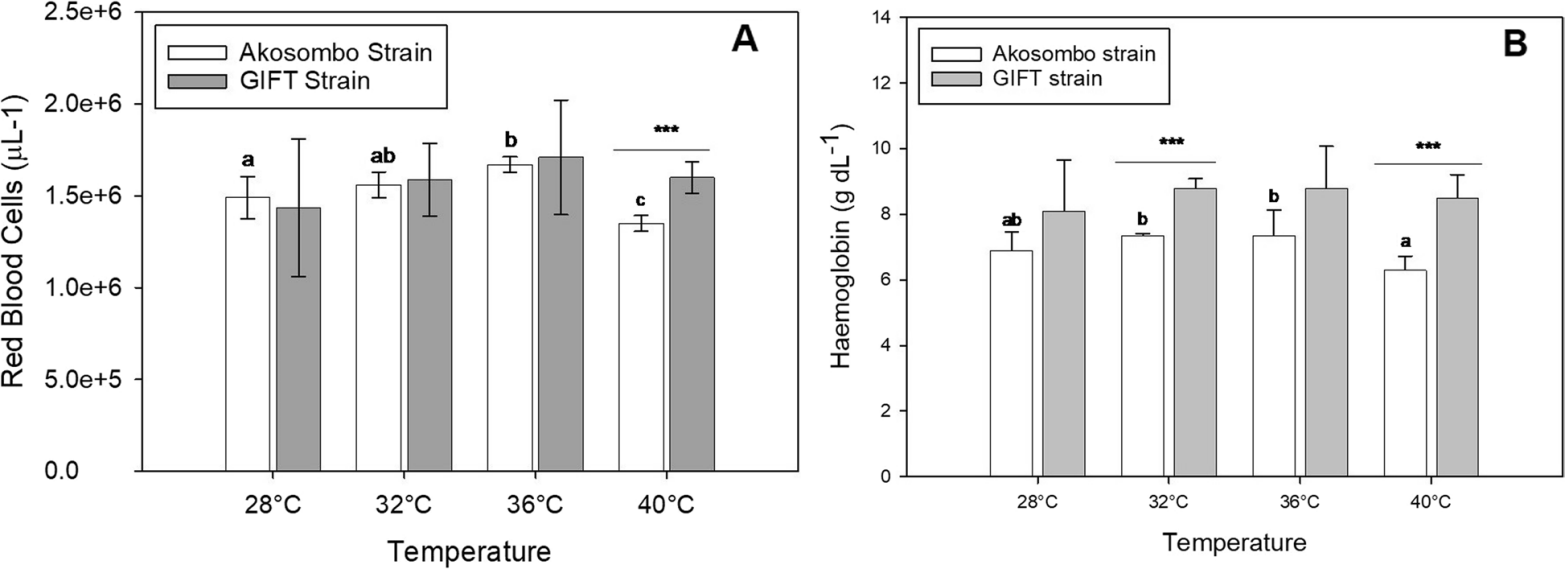
Red blood cell counts (**A**) and haemoglobin levels (**B**) of the Akosombo and GIFT strains of the Nile tilapia after exposures to progressively increasing water temperatures. Alphabets denote significant differences or otherwise for a particular strain at the different experimental temperatures. Bars at each temperature with different alphabets are significantly different from each other (*p* < 0.05). Asterisks (*) denote levels of significance between the Akosombo and GIFT strains at a specific temperature

**Fig. 6:**
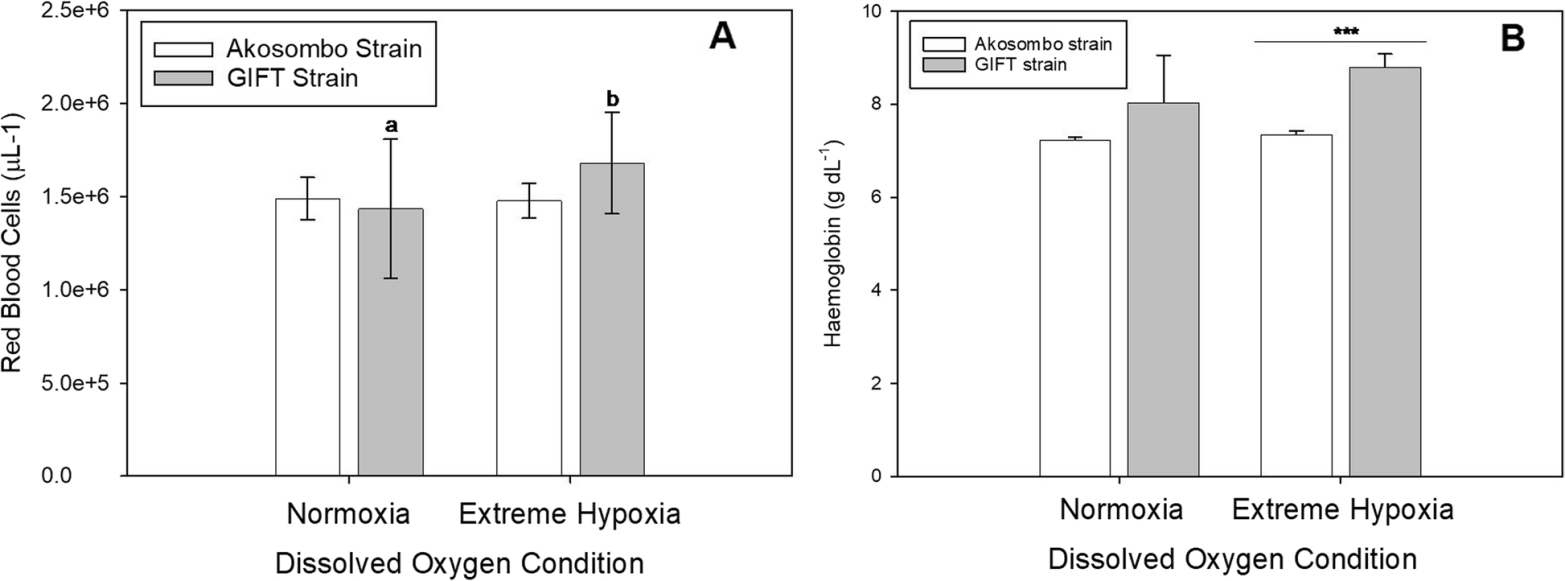
Red blood cell counts (**A**) and haemoglobin levels (**B**) of the Akosombo and GIFT strains of the Nile tilapia after exposures to normoxia and extreme hypoxia. Alphabets denote significant differences or otherwise for a particular strain at the different dissolved oxygen conditions. Bars at each dissolved oxygen concentration with different alphabets are significantly different from each other (*p* < 0.05). Asterisks (*) denote levels of significance between the Akosombo and GIFT strains at a specific oxygen condition

Exposing the two strains to incremental water temperatures up to 36 ℃ increased Red Blood Cell (RBC) counts after which the counts dropped for the two strains at 40 ℃ (Fig. 5A). Although there were elevations in RBC counts in the GIFT strain up to 36 ℃, the elevated counts did not differ significantly from the baseline level at 28 ℃. At 36 °C, the Akosombo strain had significantly higher (*p* < 0.05) RBC counts compared to the baseline levels. At 40 ℃, there was significant downregulation of RBC count in the Akosombo strain, which was significantly lower (*p* < 0,05) than the RBC counts of the GIFT strain. The different levels of hyperthermia elicited higher RBC counts in the GIFT than in the Akosombo strain. At 28 °C, the mean RBC counts of the GIFT and Akosombo strains were 1.49 ± 0.11 and 1.44 ± 0.37 × 10^6^ μL^−1^ respectively, compared to 1.67 ± 0.42 and 1.71 ± 0.31 μL^−1^ for the two strains at 36 ℃. Similar to the RBC counts, the mean haemoglobin levels of the two strains increased with increasing water temperatures up to 36 ℃ followed by depressions in levels at 40 ℃ (Fig. 5B). At 40 ℃, the marked depressions in haemoglobin counts resulted in significantly lower levels (*p* < 0.05) in the Akosombo strain than in the GIFT strain. However, the increments in haemoglobin levels within the GIFT strain were not statistically distinguishable (*p* > 0.05). There were no significant differences (*p* > 0.05) in the baseline RBC counts and the counts following the 4-h exposure to extreme hypoxia. Exposing the GIFT strain to extreme hypoxic conditions, however, resulted in significant elevations (*p* < 0.05) in RBC counts (Fig. 6A). The upregulated mean RBC count of the GIFT strain following exposure to prolonged hypoxia was significantly higher (*p* < 0.05) than the mean count of the Akosombo strain. Exposure to hypoxic conditions resulted in elevated haemoglobin levels in the two strains. The post-hypoxia exposure haemoglobin level of the GIFT strain was significantly higher (*p* < 0.05) than the level at normoxia. There were, however, significant differences (*p* < 0.05) in post-hypoxia exposure haemoglobin levels between the two strains (Fig. 6B).

### Thermal Tolerance

**Fig. 7:**
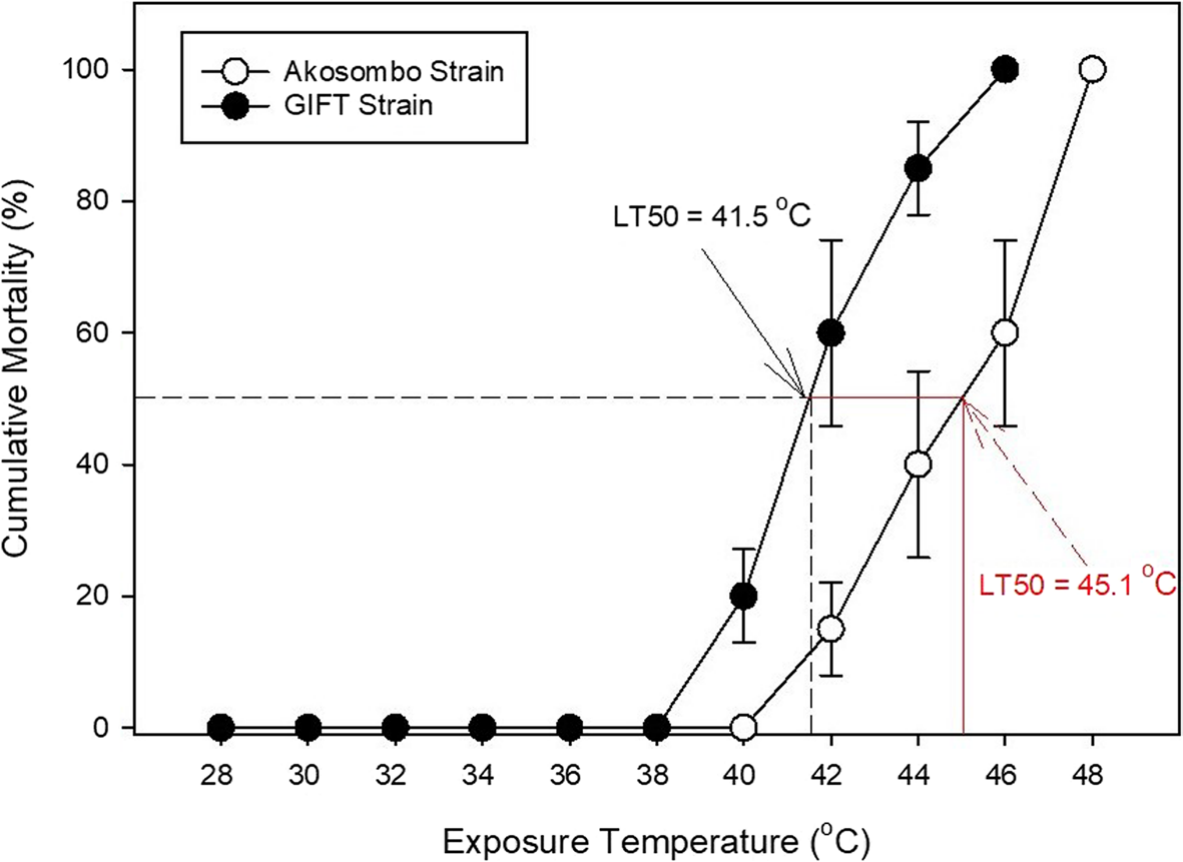
Thermal tolerance limits of the Akosombo and GIFT strains of Nile tilapia. The solid red line and black dashed line denote the upper LT_50_ values for the Akosombo and GIFT strains, respectively

The thermal tolerance trial indicated that the Akosombo strain of *O. niloticus* has higher thermotolerance than the GIFT strain. The incipient lethal temperature at which 50% mortality occurred (LT_50_) was 3.6 ℃ lower for the GIFT strain than the Akosombo strain. The LT_50_ for the GIFT and Akosombo strains were 41.5℃ and 45.1 ℃, respectively (Fig. 7). Similar to the LT_50_, LT_max_ varied markedly among the two strains. The LT_max_ for the GIFT and Akosombo strains estimated at warming rates of 0.1 ℃ min^−1^ were 46 ℃ and 48℃, respectively.

## Discussion

### Adaptation to Hypoxia and Hyperthermia Stress

For aquaculture purposes, understanding the thermal physiology of a species or strain can be essential for predicting how they will fare under culture conditions. Being ectotherms, temperature influences metabolic rates and growth is affected as it increased towards upper thermal tolerance limits (Katersky and Carter, 2007). In addition to temperature-induced elevated metabolic rates, fish must also contend with decreasing oxygen solubilities associated with increased water temperatures. In this study, the GIFT strain of Nile tilapia had significantly elevated *f*V in response to hypoxia and hyperthermia. This is probably due to higher metabolic rates of the faster-growing GIFT strain, which is directly linked to oxygen uptake. Higher metabolic rates adversely affect fish in instances of exposure to environmental stressors and lower overall fitness (Yu et al. 2021). With many aquaculture cages in Africa installed in nearshore sections of natural waters that are either eutrophic or vulnerable to several environmental changes (Musinguzi et al. 2019), this might limit the production efficiency of the GIFT strain. The lower *f*V of the Akosombo strain under normoxic and optimum temperature regimes, also indicates a comparatively lower metabolism compared to the GIFT. Similar to this study, European perch inhabiting warmer waters had lower routine metabolism than counterpart species living in colder water. Perhaps, the lower metabolism of the Akosombo strain can be linked to its long-term acclimation to warmer temperatures compared to the GIFT strains that are generally sourced from locations with colder average temperatures. Several studies have indicated positive relationships between acclimation temperature and thermal tolerance limits (e.g., Chatterjee et al. 2004; Sarma et al. 2010; Fernando et al. 2016; Ashaf-Ud-Doulah et al. 2020; Zhu et al. 2022). While thermal acclimation is generally known to improve thermal tolerance in fish, long-term acclimation to warmer temperatures can specifically improve oxygen consumption under acute temperature increases (Schulte et al. 2011). The significantly lower *f*Vs of the Akosombo strain at 40 ℃ is perhaps reflective of higher metabolic compensatory mechanisms. Wide adjustments in metabolic capacities in fish to compensate for the effects of thermal shocks is critical to adapting to environmental temperature changes (Bennet et al. 2021).

In this study, mortality was first recorded for the GIFT strain when the water temperature exceeded 38 ℃. This is consistent with the findings of Qiang et al. (2013) that reported that GIFT strains experience mortality when water temperature exceeds 37 °C. The higher thermal tolerance of the local Nile tilapia strain mirrors the lethal temperature of ≥ 40 °C reported by Abass et al. (2018). While the actual mechanisms for the higher hyperthermia tolerance in the Akosombo strain remain unclear, gill morphology and heat shock proteins might have played key roles in determining fitness following exposure to high temperatures. Inhibition of aerobic metabolism under elevated water temperatures can also affect fish survival (Zhou et al. 2022). Although compared to most cultured fish species, the GIFT strain of the Nile tilapia has higher upper thermal limits, it is possible that the forecasted progressive global warming will affect GIFT culture. The combinations of lower hypoxia and thermal tolerances, compared to its native counterpart, might threaten the overall fitness of the culture strain over a long temporal scale. Therefore, although the GIFT strain exhibits greater growth performance in the short term, the Akosombo strain stands a better chance of survival or delayed mortality in the event of extreme hyperthermia and hypoxia. Despite the higher resilience of the Akosombo strain to environmental stressors, it is also plausible that pre-experiment acclimation conditions in the different hatcheries had some impact on the observed results. It is possible to hypothesize that some of the variations in aerobic performance and tolerance to acute hypoxia and heat stress could have been explained by variations in O_2_ and temperature levels the different strains were exposed to in their respective hatcheries.

### Gill Ventilation Frequency and Aquatic Surface Respiration

This study recorded significantly elevated gill ventilation frequencies in the two Nile tilapia strains following exposures to progressive hypoxia. Ventilation frequencies increased linearly with declining O_2_ levels up to an acute hypoxia threshold beyond which there were downregulated ventilatory responses. Fish under stressful conditions typically exhibit hyperventilation (Kramer 1987; Perry et al. 2009; Obirikorang et al. 2020). However, individuals of the GIFT strain exhibited significantly higher *f*Vs compared to the Akosombo strains under normoxia and moderate hypoxia. This possibly points to the lower hypoxia tolerance of the strain. Hyper-ventilatory responses in fish represent energetic costs that can potentially divert energy away from growth and reduce the overall fitness of an organism (Perry et al. 2009). The GIFT strain’s significantly higher gill ventilation frequencies following hypoxia and hyperthermia stressors could have played key roles in their lower tolerances of low dissolved oxygen at high temperatures. Hypoxic ventilatory responses for both strains increased with decreasing O_2_ levels peaking at 1.8 mg L^−1^; below this threshold, the *f*V decreased for both groups of fish. Fish under chronic hypoxia may either respond by actively trying to maintain the O_2_ uptake rate (oxyregulation), or by downregulating O_2_ demand and uptake (oxyconformation) (Perry et al. 2009). The exhibition of oxyregulation and oxyconformation by Nile tilapia over a wide dissolved oxygen range has been reported in a study by Obirikorang et al. (2020). The reductions in *f*V below the critical oxygen threshold could have been because hyperventilation became exhaustive and counterproductive over time, forcing fish to resort to oxyconformation. Oxyconformation, or in extreme cases, cessation of ventilatory behaviour are adaptive behavioural mechanisms by fish to reduce the environmental exposure of gill epithelia and extend tolerance limits (King and Sardella 2017).

The ASR trials showed strain-specific differences in the threshold at which ASR was employed. The Akosombo strain exhibited a higher physiological plasticity during the ASR trials where the strain generally performed ASR at lower O_2_ thresholds than the GIFT strain. Aquatic surface respiration is an adaptive mechanism that reduces the additional cost of ventilation during hypoxia (Kramer 1987). Unsurprisingly, the Akosombo strain did not employ this strategy until below 1 mg L^−1^. Conversely, ASR was first performed by the GIFT strain at a DO level over two-fold higher (2.17 mg L^−1^) than in the Akosombo strain. Morphological and physiological variables coupled with several genes and metabolic pathways usually underlie intraspecific differences in hypoxia tolerance among fishes (Richards 2011).

### Effects of Hypoxia and Hyperthermia on Haematology

Evidence from several studies indicate that environmental stressors either upregulate or downregulate haematological markers such as haemoglobin and RBC in fish (Obirikorang et al. 2019; Sheng et al. 2019; Dagoudo et al. 2021). The exposures of the two strains of Nile tilapia to acute hyperthermia and hypoxia resulted in upregulations in both haemoglobin and RBC, at least up to a point. Increment in RBCs under hyperthermia is directly linked to the acceleration of respiratory metabolism and the higher oxygen demand (Bao et al. 2018). Increment in RBC levels under elevated temperatures is a compensatory response to ensure an enhanced uptake and delivery of oxygen, and has been reported in the rainbow trout (*Oncorhynchus mykiss*) (Dewilde and Houson 1967) and the Nile tilapia (*O. niloticus*) (Bao et al. 2018). There were downregulations of RBC and haemoglobin at 40 ℃ in both strains. Acute stress can downregulate RBC adrenergic responses in some fish species (Gilmour et al. 1994).

Exposing the GIFT strain to extreme hypoxia conditions resulted in significantly elevated RBC counts (*p* < 0.05) compared to baseline levels. The splanchnic release of erythrocytes to increase RBC counts under low oxygen partial pressures as an adaptive response to counteract oxygen crises is common in fish (Nikinmaa and Boutilier 1995). Under acute hypoxic stress, an increment in RBC parameters increases the oxygen‐binding capacity of the blood and improves oxygen utilization (Sheng et al. 2019).

## Conclusion

The study examined the tolerance limits (ASR, LT_max_) of the native and foreign strains of Nile tilapia following exposures to hyperthermic and hypoxic stressors and the effects of these stressors on some haematological parameters and adaptive behavioural responses. The Akosombo strain had significantly higher lethal temperature endpoints and exhibited lower ventilation frequencies at temperatures higher than its thermoneutral temperature compared to the GIFT strain. To counteract hypoxic shocks, the Akosombo strain were less susceptible to hypoxia and performed ASR at substantially lower dissolved oxygen levels. Compared to the GIFT strain, they also showed relatively smaller ventilatory adjustments to counteract the hypoxic effects. These findings suggest that it is important to consider the effects of environmental conditions and changes on the performance of the GIFT strain during culture. To offset the deleterious effects of high temperature and low dissolved oxygen levels, fish farmers, especially those who culture the GIFT strains can schedule production cycles to coincide with cooler months when ambient temperatures are within the optimum ranges and dissolved oxygen levels higher. This strategy might also reduce incidences of pathogen-induced tilapia mortalities that are generally exacerbated in tropical aquaculture during the warmer periods. While overall the Akosombo strain had higher stress tolerance, the possible effects of the pre-trial hatchery habituations of the different strains on fish performance under the different stress trials should not be discounted. However, by testing the tolerance of the two strains to extreme environmental conditions, and measuring some physiological and behavioural perturbations, this study provides important benchmarking data that complements the comparative growth study of the two strains conducted by Trinh et al. (2021). The study expands the GIFT strain performance trials necessary for its adoption in Africa. Future trials can, however, focus on molecular examinations such as oxygen-dependent gene expression and heat shock protein expression.

## Materials and methods

### Experimental Animals and Conditioning

Two hundred sex-reversed male *O. niloticus* sub-adults (average weight: 180.1 ± 3.1 g) of each strain, grown in non-aerated outdoor concrete tanks at two commercial hatcheries, were used for the trials. The Akosombo strain was sourced from the Aquaculture Development Centre, a government hatchery in southern Ghana, while the GIFT strain (a foreign strain) was obtained from a private hatchery in south-eastern Ghana (280 km apart). Following transportation from their hatcheries, individuals of the same strain were conditioned together in 130 L thermoplastic PETG tanks of a recirculating aquaculture system at a rate of 7 fish m^−3^ for two weeks. During the conditioning period, water was continuously cycled from a 1000 L reservoir tank into the culture tanks at a regulated rate of 100 L h^−1^ by a submersible pump (Aqua Forte DM-1300, Sibo Fluidra B.V., Doornhoek, Netherlands). Outflowing water from the culture tanks was treated using a mechanical filter and a biofilter system filled with four bio-blocks, each with a surface area of 150 m^2^m^−3^, before cycling back into the culture tanks. Fish were kept under constant normoxia (O_2_ concentration: 6.5 mg L^−1^) and hand-fed with a commercial extruded diet (Raanan Tilapia Supreme Growth Feed (2.5 mm pellet size), 33% crude protein, 5% lipid) at a rate of 3% of total fish biomass. The acclimation temperature was 28.3 ± 1.1 ºC. After the acclimation period, fish were transferred in batches into different 60 L tanks and exposed to various stress conditions.

### Oxygen Consumption (*MO*_2_) Measurements

The closed respirometry method, which involved putting nearly-equal masses of each Nile tilapia strain in a sealed chamber of known volume and measuring respiration-driven O_2_ decline at various times throughout the experiment, was used to estimate *M*O_2_ in this study. For each *MO*_*2*_ run, ten similar-sized fish of each strain (Akosombo strain: 180.22 ± 1.20 g; GIFT strain: 179.7 ± 1.34 g) were transferred into two 60L chambers fitted with a calibrated oxygen probe (Hach HQ40d, CO, USA) pre-programmed to record O_2_ levels every 5 min. Before and after each *M*O_2_ run, oxygenated water was circulated from a sump containing using a submersible pump (Aqua Forte DM-1300 Sibo Fluidra B.V, Doornhoek, Netherlands) before cycling back into the sump. Fish were fasted for 24-h before they were transferred into the respirometry chamber and acclimated for an additional 24-h. During the acclimation period, fish were kept under normoxic conditions (O_2_ concentration: 6.7 ± 0.2 mg L^−1^). After the acclimation period, the chamber was sealed and aeration stopped, allowing the fish's respiration to reduce the oxygen levels. Each group was kept in the chamber until oxygen saturation reached 0.7 mg L^−1^. Once this hypoxic point was reached, the run was terminated and normoxic water flushed through the chamber. In all the runs, extreme hypoxia was achieved within 121 ± 6 min. No fish experienced loss of equilibrium (LOE) or died during the experiments. Before each measurement run, the tanks were thoroughly cleaned to remove all organic substances that might contribute to oxygen consumption. Additionally, two sham *MO*_*2*_ runs with no fish in the tanks were performed to check for air exchange and microbial consumption, which were used to correct *MO*_*2*_. The *Ṁ*O_2_ measurements were performed by linear analysis of the rate of oxygen decline (ΔpO_2_ Δt^−1^) in each tank, as described by the equation:

*MO*_*2*_ = *αV*_*tank*_*βBM*^*−1*^ (Steffensen et al. 1984);

Where *ṀO*_*2*_ is the mass-specific oxygen consumption rate (mg O_2_ kg^−1^ h^−1^), α is the slope (ΔpO_2_ Δt^− 1^), V_tank_ is the volume of the experimental tank minus the volume of the fish, BM is the fish body mass in kilograms, and β is oxygen solubility at a given temperature. The trials for each strain were run in triplicate.

### Aquatic Surface Respiration (ASR)

Thirty individuals of each strain (average mass: 181.20 ± 2.54 g) were assigned in triplicate groups at a rate of 10 fish per tank into 60L transparent tanks half-filled with water. To achieve the levels of hypoxia required to trigger ASR, the air supply to each tank was shut off, allowing the fish to consume the oxygen in the culture water. The similar body masses ensured that oxygen decline rates were fairly similar in each run, and extreme hypoxia level of 0.2 mg L^−1^ O_2_ was reached in 192 ± 10 min. Additionally, the surface of each tank was completely covered with a piece of polythene sheet to minimize gas exchange at the water surface. Aquatic surface respiration (ASR) thresholds were measured by exposing the fish to the progressively decreasing DO levels (6.5—0.2 mg L^−1^ O_2_) and estimating the DO level at which 1 (ASR_10_), 3 (ASR_30_), 5 (ASR_50_), 7 (ASR_70_), and 9 (ASR_90_) out of the ten fish per tank performed ASR. ASR in this study was identified as fish maintaining a position at the surface of the tank and ventilating their gills with their mouths touching the uppermost layer of the water for several seconds (Chapman and McKenzie, 2009). Each tank was fitted with a calibrated oxygen probe (Hach HQ40d, CO, USA) pre-programmed to log dissolved oxygen levels every 5 min during exposure and track O_2_ levels and rates of decline. Each tank was shielded on all sides except the front with cardboard to minimize fish disturbance during counts of ASR.

### Ventilation Frequency (*f*V) Measurements

Separate groups of 10 fish per strain were transferred into 60-L aquarium tanks containing 30 L of water and subjected to progressively declining O_2_ levels using the approach described for the ASR measurements and *f*V recorded. Counts of *f*V were also recorded for a group of 10 fish per strain exposed to increasing water temperatures from 28 to 40 ℃ (+ 0.1 ℃ per minute). Ventilation frequency was recorded by counting the number of opercular movements over a 10 s period and normalizing the data to 1 min. Following the initial measurements at 28 ℃, gill ventilation frequencies were recorded after every 4 ℃ rise in temperature. Water temperatures were regulated using a 200 W immersion heater connected to a temperature controller (Aqua Medic T controller HC, Bissendorf, Germany) set to the upper exposure limit. The measurements of gill ventilation frequencies in relation to hypoxia and hyperthermia were each run with triplicate groups of each strain. At each O_2_ and temperature threshold, *f*V was recorded as the number of gill ventilations occurring in 15-s periods and extrapolated for 1 min. Heaters were turned off at the set upper temperature limit once gill ventilation recordings were completed, and water was allowed to cool naturally at room temperature. All fish groups were fasted for 24 h before each trial.

### Hyperthermia or Hypoxia Effects on Haematological Parameters

Separate groups of fasted fish were exposed to progressively increasing levels of hyperthermia or hypoxia.using the same approaches described for the thermal-induced *f*V and ASR trials. In each trial, triplicate groups of 10 fish per tank were used, and the effects of either hyperthermia or hypoxia on haematological parameters assessed. Before exposure to the increasing temperatures, blood was sampled from an initial group of 6 fish per treatment at the initial temperature of 28℃. For the hyperthermia stress test, two fish per tank (*n* = 6 per strain) were carefully netted from each tank after every temperature increment of 4 ℃ until water temperature reached 40 ℃. Fish were then anaesthetized in aerated water containing propofol (Fresenius Kabi AB, Uppsala, Sweden) at a concentration of 100 mg L^−1^. To mitigate the possibility of netting provoking acute stress, fish were allowed to gently swim into scoop nets placed in their swimming paths and gently transferred into an adjacent anaesthetic bath. About 2 ml of blood sample was drawn from the caudal vasculature of each fish into separate Vacutainer® tubes (BD Vacutainer, catalogue number 367861; Becton Dickinson, Franklin Lakes, NJ, USA) containing EDTA as anticoagulant for haematological analysis. After sampling, fish were placed in tanks of aerated fresh water for recovery. The effect of hypoxia stress on haematological parameters was tested in groups of each strain from the acclimation group subjected to constant normoxia (~ 6.5 mg L^−1^ O_2_) and a separate group subjected to acute hypoxia (0.2 mg L^−1^) for 4 h. Blood was drawn from 3 fish per tank following the same procedure described above.

### Haematological Analysis

Collected blood samples were immediately sent to the laboratory for haematological analyses using the automated blood cell count method (Tran-Duy et al. 2008; Fazio et al. 2012). The haematological analyses were carried out as complete red blood cell (RBC) counts and haemoglobin concentrations using an automated haematology analyzer (CELL-DYN 1800, Abbot Laboratories, Irving, TX, USA). All samples were analysed in duplicate within two hours of collection.

### Thermal Tolerance Trials

The maximum lethal temperature (LT_max_) was used as an index of thermal tolerance for the Akosombo and GIFT strains of *O. niloticus*. For each LT_max_ trial, individuals of each strain (*n* = 10) were transferred into 60 L tanks with initial water temperatures matching the acclimation temperature. Each group was habituated to their new environment for 24 h before the trials, during which they were not fed. The trials were run in triplicate for each strain. During the LT_max_ trials water temperature was increased by 0.1 °C min^−1^ from 28 ℃ until all fish reached lethal endpoints. Fish were considered to have reached a lethal endpoint when it remained dorsally recumbent with complete cessation of opercular movements (Ciji et al. 2021). Tanks were aerated throughout the trials to maintain appropriate levels of normoxia. Small recirculating water pumps (1000, Eheim, Germany) were placed in the middle of the water column within each tank to circulate water and ensure uniform heating radially.

### Statistical Analysis

Data were expressed as mean ± standard deviation. Before comparing the two strains, data were subjected to Kolmogorov–Smirnov and Bartlett's tests to test for normality and homoscedasticity, respectively. The data complied with the assumption of parametric tests, thus unpaired Student’s t-test was used to test for differences in the means of measured parameters between the two *O. niloticus* strains. Within the same strain, the effect of the different water temperatures on RBC and haemoglobin levels were tested using one-way analysis of variance (one-way ANOVA). In instances where there were significant differences among the treatment means, a post hoc test was conducted using the Tukey multiple comparisons test (Zar, 1999). In all cases, means were considered statistically significant at *p* < 0.05. All statistical analyses and graphs were executed using SigmaPlot ver. 12.0 (Systat Software Inc, San Jose, CA, USA).

## Data Availability

The data that support the findings of this study are available from the corresponding author, upon reasonable request.

## Abbreviations

ANOVA: Analysis of variance
ASR: Aquatic surface respiration
EDTA: Ethylenediaminetetraacetic acid
*f*v: Gill ventilations frequencies
GIFT: Genetically improved farmed tilapia
LT_50_: Median lethal temperature
LT_max_: Maximum lethal temperature
*MO*_*2*_: Oxygen consumption rate
O_2_: Oxygen
RBC: Red blood cells
